# A Green Strategy for Federated and Heterogeneous Clouds with Communicating Workloads

**DOI:** 10.1155/2014/273537

**Published:** 2014-11-11

**Authors:** Jordi Mateo, Jordi Vilaplana, Lluis M. Plà, Josep Ll. Lérida, Francesc Solsona

**Affiliations:** ^1^Department of Computer Science & INSPIRES, University of Lleida, Jaume II 69, 25001 Lleida, Spain; ^2^Department of Mathematics, University of Lleida, Jaume II 73, 25001 Lleida, Spain

## Abstract

Providers of cloud environments must tackle the challenge of configuring their system to provide maximal performance while minimizing the cost of resources used. However, at the same time, they must guarantee an SLA (service-level agreement) to the users. The SLA is usually associated with a certain level of QoS (quality of service). As response time is perhaps the most widely used QoS metric, it was also the one chosen in this work. This paper presents a green strategy (GS) model for heterogeneous cloud systems. We provide a solution for heterogeneous job-communicating tasks and heterogeneous VMs that make up the nodes of the cloud. In addition to guaranteeing the SLA, the main goal is to optimize energy savings. The solution results in an equation that must be solved by a solver with nonlinear capabilities. The results obtained from modelling the policies to be executed by a solver demonstrate the applicability of our proposal for saving energy and guaranteeing the SLA.

## 1. Introduction

In cloud computing, SLA (service-level agreement) is an agreement between a service provider and a consumer where the former agrees to deliver a service to the latter under specific terms, such as time or performance. In order to comply with the SLA, the service provider must monitor the cloud performance closely. Studying and determining SLA-related issues are a big challenge [1, 2].

GS is designed to lower power consumption [3] as much as possible. The main objective of this paper is to develop resource scheduling approaches to improve the power efficiency of data centers by shutting down and putting idles servers to sleep, as Intel's Cloud Computing 2015 Vision [4] does.

At the same time, GS is aimed at guaranteeing a negotiated SLA and power-aware [3] solutions, leaving aside such other cloud-computing issues as variability [2], system security [3], and availability [5]. Job response time is perhaps the most important QoS metric in a cloud-computing context [1]. That is also why the QoS parameter is chosen in this work. In addition, despite good solutions having been presented by some researchers in the literature dealing with QoS [6, 7] and power consumption [8, 9], the model presented aims to obtain the best scheduling, taking both criteria into account.

This paper is focused on proposing a static green alternative to solving the scheduling problem in cloud environments. Many of the cited solutions consist of creating dynamically ad hoc VMs, depending on the workload, made up of independent tasks or a parallel job composed of communicating or noncommunicating tasks. This implies constantly creating, deleting, or moving VMs. These processes consume large amounts of time. Low ratios for return times and VM management should lead to proposing more static scheduling methods between the existing VMs. The solution described in this paper goes on this direction. However, this solution can be merged and complemented with dynamic proposals.

Our additional contribution with respect to [10] is that our solution tries to optimize 2 criteria at the same time: scheduling tasks to VMs, saving energy, and consolidating VMs to the nodes. Providing an efficient NLP solution for this problem is a novelty challenge in the cloud computing research field.

Another important contribution of this paper is method used to model the power of the virtual machines in function of their workload. Relying on the work done in [11], where the authors formulate the problem of assigning people from various groups to different jobs and who may complete them in the minimum time as a stochastic programming problem, the job completion times were assumed to follow a Gamma distribution. To model the influence of the workload, the computing power of the virtual machine is weighted by a load factor determined by an* Erlang* distribution (equivalent to a Gamma). Finally, a stochastic programming problem is obtained and transformed into an equivalent deterministic problem with a nonlinear objective function.

The remainder of the paper is organized as follows. Our contribution is based on the previous work presented in Section 2. In the GS section (Section 3), we present our main contributions, a sort of scheduling policy. These proposals are arranged by increasing complexity. The experimentation showing the good behavior of our cloud model is presented in the Results section (Section 4). Finally, the Conclusions and Future Work section outlines the main conclusions and possible research lines to explore in the near future.

## 2. Related Work

There is a great deal of work in the literature on linear programming (LP) solutions and algorithms applied to scheduling, like those presented in [12, 13]. Another notable work was performed in [14], where authors designed a Green Scheduling Algorithm that integrated a neural network predictor in order to optimize server power consumption in cloud computing. Also, the authors in [15] proposed a genetic algorithm that takes into account both makespan and energy consumption.

Shutting down servers when they are not being used is one of the most direct methods to reduce the idle power. However, the authors in [16] state that a power-off requires an additional setup cost, resulting in long system delays. Shutting down servers may sacrifice quality of service (QoS) levels, thus violating the SLA. They put the server work at a lower service rate rather than completely stopping work during idle periods. This drawback can be reduced if scheduling is performed for a large enough number of tasks, as in our case.

In [17], the authors treat the problem of consolidating VMs in a server by migrating VMs with steady and stable capacity needs. They proposed an exact formulation based on a linear program described by too small a number of valid inequalities. Indeed, this description does not allow solving, in a reasonable time or an optimal way, problems involving the allocation of a large number of items (or VMs) to many bins (or servers).

In [18], the authors presented a server consolidation (Sercon) algorithm which consists of minimizing the number of used nodes in a data center and minimizing the number of migrations at the same time to solve the bin (or server) packing problem. They show the efficiency of Sercon for consolidating VMs and minimizing migrations. Despite our proposal (based on NLP) always finding the best solution, Sercon is a heuristic that cannot always reach or find the optimal solution.

The authors in [19] investigated resource optimization, service quality, and energy saving by the use of a neural network. These actions were specified in two different resource managers, which sought to maintain the application's quality service in accordance with the SLA and obtain energy savings in a virtual servers' cluster by turning them off when idle and dynamically redistributing the VMs using live migration. Saving energy is only applied in the fuzzy-term “*intermediate load*,” using fewer resources and still maintaining satisfactory service quality levels. Large neural network training times and their nonoptimal solutions could be problems that can be overcome by using other optimization techniques, such as the NLP one used in this paper.

In [10], the authors modelled an energy aware allocation and consolidation policies to minimize overall energy consumption with an optimal allocation and a consolidation algorithm. The optimal allocation algorithm is solved as a bin-packing problem with a minimum power consumption objective. The consolidation algorithm is derived from a linear and integer formulation of VM migration to adapt to placement when resources are released.

The authors of [20] presented an effective load-balancing genetic algorithm that spreads the multimedia service task load to the servers with the minimal cost for transmitting multimedia data between server clusters and clients for centralized hierarchical cloud-based multimedia systems. Clients can change their locations, and each server cluster only handled a specific type of multimedia task so that two performance objectives (as we do) were optimized at the same time.

In [21], the authors presented an architecture able to balance load into different virtual machines meanwhile providing SLA guarantees. The model presented in that work is similar to the model presented in this paper. The main difference is in the tasks considered. Now, a more complex and generalized model is presented. In addition, communicating and heterogeneous tasks as well as nondedicated environments have been taken into account.

Our proposal goes further than the outlined literature. Instead of designing a single criteria scheduling problem (LP), we design an NLP scheduling solution which takes into account multicriteria issues. In contrast to the optimization techniques of the literature, our model ensures the best solution available. We also want to emphasize the nondedicated feature of the model, meaning that the workload of the cloud is also considered. This also differentiates from the related work. This consideration also brings the model into reality, providing more reliable and realistic results.

## 3. GS Model

The NLP scheduling solution proposed in this paper models a problem by giving an objective function (OF). The equation representing the objective function takes various performance criteria into account.

GS tries to assign as many tasks as possible to the most powerful VMs, leaving the remaining ones aside. As we will consider clouds made up of various nodes, at the end of the scheduling process, the nodes all of whose VMs are not assigned any task can then be turned off. As SLA based on the minimization of the return time is also applied, the model also minimizes the computing and communication time of the overall tasks making up a job.

### 3.1. General Notation

Let a job made up of *T* communicating tasks (*t* ^*i*^,  *i* = 1,…, *T*), and a cloud made up of *N* heterogeneous nodes (Node_1_,…, Node_*N*_).

The number of VMs can be different between nodes, so we use notation *v*_*n*_ (*v*_*n*_ = 1,…, *V*_*n*_, where *n* = 1,…, *N*) to represent the number of VMs located to Node_*n*_. In other words, each Node_*n*_ will be made up by VMs VM_*n*1_,…, VM_*nV*_*n*__.

Task assignments must show the node and the VM inside the nodes task *t* ^*i*^ is assigned to. In doing so, Boolean variables will also be used. The notation *t*_*nv*_*n*__^*i*^ is used to represent the assignment of task *t* ^*i*^ to Node_*n*_ VM VM_*nv*_*n*__.

The notation *M*_*nv*_*n*__^*i*^ represents the amount of* Memory* allocated to task *t* ^*i*^ in VM VM_*nv*_*n*__. It is assumed that Memory requirements do not change between VMs, so *M*_*nv*_*n*__^*i*^ = *M*_*nv*_*n*_′_^*i*^  ∀*n* ≤ *N*, and *v*_*n*_, *v*_*n*_′ ≤ *V*_*n*_. The Boolean variable *t*_*nv*_*n*__^*i*^ represents the assignment of task *t* ^*i*^ to VM_*nv*_*n*__. Once the solver is executed, the *t*_*nv*_*n*__^*i*^ variables will inform about the assignment of tasks to VMs. This is *t*_*nv*_*n*__^*i*^ = 1 if *t* ^*i*^ is assigned to VM_*nv*_*n*__, and *t*_*nv*_*n*__^*i*^ = 0 otherwise.

### 3.2. Virtual Machine Heterogeneity

The* relative computing power* (Δ_*nv*_*n*__) of a VM_*nv*_*n*__ is defined as the* normalized score* of such a VM. Formally, consider

(1) $$\begin{array}{c} \Delta_{nv_{n}}=\frac{\delta_{nv_{n}}}{\sum_{i=1}^{V}\sum_{k=1}^{V_{i}}\delta_{iv_{k}}}, \end{array}$$

where ∑_*i*=1_^*V*^∑_*k*=1_^*V*_*i*_^Δ_*iv*_*k*__ = 1. *δ*_*nv*_*n*__ is the score (i.e., the computing power) of VM_*nv*_*n*__. Although *δ*_*nv*_*n*__ is a theoretical concept, there are many valid benchmarks it can be obtained with (i.e., Linpack (Linpack. http://www.netlib.org/linpack/) or SPEC (SPEC. http://www.spec.org)). Linpack (available in C, Fortran and Java), for example, is used to obtain the number of floating-point operations per second. Note that the closer the* relative computing power* is to one (in other words, the more powerful it is), the more likely it is that the requests will be mapped into such a VM.

### 3.3. Task Heterogeneity

In order to model task heterogeneity, each task *t* ^*i*^ has its* processing cost P*_*nv*_*n*__^*i*^, representing the execution time of task *t* ^*i*^ in VM_*nv*_*n*__ with respect to the execution time of task *t* ^*i*^ in the least powerful VM_*nv*_*n*__ (in other words, with the lowest Δ_*nv*_*n*__). It should be a good choice to maximize *t*_*nv*_*n*__^*i*^ *P*_*nv*_*n*__^*i*^ to obtain the best assignment (in other words, the OF) as follows:

(2) $$\begin{array}{c} \max\left(\overset{N}{\underset{n=1}{\sum }}\;\overset{V_{n}}{\underset{v_{n}=1}{\sum }}\;\overset{T}{\underset{i=1}{\sum }}t_{nv_{n}}^{i}P_{nv_{n}}^{i}\right). \end{array}$$

However, there are still a few criteria to consider.

### 3.4. Virtual Machine Workload

The performance drop experienced by VMs due to workload saturation is also taken into account. If a VM is underloaded, its throughput (tasks solved per unit of time) will increase as more tasks are assigned to it. When the VM reaches its maximum workload capacity, its throughput starts falling asymptotically towards zero. This behavior can be modeled with an* Erlang* distribution density function.* Erlang* is a continuous probability distribution with two parameters, *α* and *λ*. The *α* parameter is called the shape parameter, and the *λ* parameter is called the rate parameter. These parameters depend on the VM characteristics. When *α* equals 1, the distribution simplifies to the exponential distribution. The* Erlang* probability density function is

(3) $$\begin{array}{c} E\left(x;\alpha ,\lambda \right)=\lambda e^{-\lambda x}\frac{{\left(\lambda x\right)}^{\alpha -1}}{\left(\alpha -1\right)!}\;\forall x,\lambda \geq 0. \end{array}$$

We consider that the* Erlang* modelling parameters of each VM can easily be obtained empirically. The* Erlang* parameters can be obtained by means of a comprehensive analysis of all typical workloads being executed in the server, supercomputer, or data center to be evaluated. In the present work, a common PC server was used. To carry out this analysis, we continuously increased the workload until the server was saturated. We collected measurements about the mean response times at each workload variation. By empirical analysis of that experimentation, we obtained the* Erlang* that better fitted the obtained behaviour measurements.

The* Erlang* is used to weight the* Relative computing power *Δ_*nv*_*n*__ of each VM_*nv*_*n*__ with its associated workload factor determined by an* Erlang* distribution. This optimal workload model is used to obtain the maximum* throughput* performance (number of task executed per unit of time) of each VM_*nv*_*n*__. In the case presented in this paper, the *x*-axis (abscisas) represents the sum of the* Processing cost P*_*nv*_*n*__^*i*^ of each *t* ^*i*^ assigned to every VM_*nv*_*n*__.

**Figure 1:**
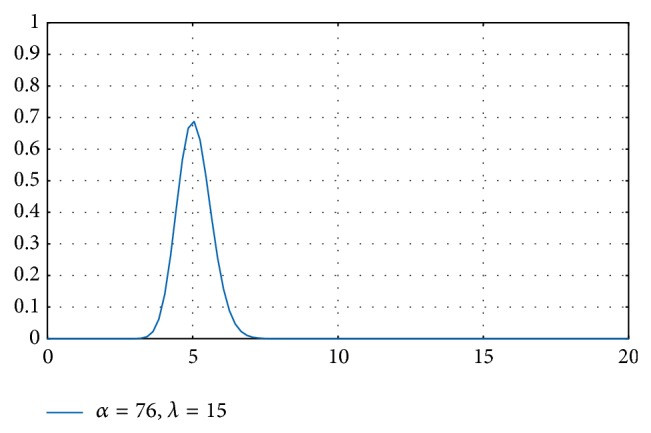
*Erlang* plots for different *α* and *λ* values.

Figure 1 shows an example in which we depict an* Erlang* with *α* = 76 and *λ* = 15. The* Erlang* reaches its maximum when *X* = 5. Provided that the abscissas represent the workload of a VM, a workload of 5 will give the maximum performance to such a VM in terms of throughput. So we are not interested in assigning less or more workload to a specific VM_*nv*_*n*__ because otherwise, this would lead us away from the optimal assignment.

Given an* Erlang* distribution function with fixed parameters *α* and *λ*, it is possible to calculate the optimal workload in which the function reaches the maximum by using its derivative function:

(4) $$\begin{array}{c} E{\left(x;\alpha ,\lambda \right)}^{\prime }=e^{-\lambda -1*x}*x\left(\lambda -1*x-\alpha -1\right)\;\forall x,\lambda \geq 0. \end{array}$$

Accordingly, the optimal workload of the* Erlang* example in Figure 1, with *α* = 76 and *λ* = 15, is

(5) $$\begin{array}{c} E{\left(x;76,15\right)}^{\prime }=e^{-14*x}*x\left(14x-75\right)=0\\ x=\frac{75}{14}. \end{array}$$

Finally, in our case, provided that the VM workload, defined as the sum of the processing costs (*P*_*nv*_*n*__^*i*^) of the tasks assigned to a particular VM, must be an *N* number, the optimal workload (*x*) is

(6) $$\begin{array}{c} x=\text{round}\left(\frac{75}{14}\right)=5. \end{array}$$

Provided that Boolean variable *t*_*nv*_*n*__^*i*^ = 1 is a boolean variable informing of the assignment of *t* ^*i*^ to VM_*nv*_*n*__, and *t*_*nv*_*n*__^*i*^ = 0 if not, the* Erlang*-weighted Δ_*nv*_*n*__ would be

(7) $$\begin{array}{c} \Delta_{nv_{n}}E\left(\overset{T}{\underset{i=1}{\sum }}P_{nv_{n}}^{i}t_{v}^{j};\alpha ,\lambda \right). \end{array}$$

### 3.5. Task Communication and VM Selection

In this section, the VM selection is also considered. In doing so, each VM can be selected from a range of *N* nodes, forming a federated cloud. We want to obtain an OF that considers the scheduling of heterogeneous and communicating tasks to *N* heterogeneous nodes made up of different numbers of heterogeneous VMs.

The* communication cost* (in time) between tasks *t* ^*i*^ and *t* ^*j*^ when in the same VM is denoted by *C* ^*ij*^ and should be passed to the solver as an argument. For reasons of simplicity, all communication links are considered to have the same bandwidth and latency. Notation *C*_*nv*_*n*__^*ij*^ represents the* communication cost* between task *t* ^*i*^ residing in VM_*nv*_*n*__ with another task *t* ^*j*^ (located in the same VM or elsewhere). Provided equivalent bandwidth between any two VMs, *C*_*nv*_*n*__^*ij*^ = *C*_*nv*_*n*_′_^*ji*^  ∀*v*, *v*′ ≤ *V*. In other words, the* communication cost* does not depend on the VM or the links used between the VMs.

VM communication links are considered with the same bandwidth capacity. Depending on its location, we multiply the communication cost between tasks *t* ^*i*^ and *t* ^*j*^ by a given* communication slowdown*. If *t*_*i*_ and *t*_*j*_ are located in the same VM, the* communication slowdown* (denoted by Cs_*nv*_*n*__) is 1. If *t* ^*j*^ is assigned to another VM in the same node than *t* ^*i*^ ($t_{n\bar{v_{n}}}^{j}=1$, the* Communication slowdown* ($\text{C}{\text{s}}_{n\bar{v_{n}}}$) will be in the range [0,…, 1]. Finally, if *t* ^*j*^ is assigned to another VM located in another node ($t_{\bar{nv_{n}}}^{j}=1$), the corresponding* communication slowdown* term ($\text{C}{\text{s}}_{\bar{nv_{n}}}$) will also be in the range [0,…, 1]. $\text{C}{\text{s}}_{n\bar{v_{n}}}$ and $\text{C}{\text{s}}_{\bar{nv_{n}}}$ should be obtained with respect to Cs_*nv*_*n*__. In other words, $\text{C}{\text{s}}_{n\bar{v_{n}}}$ and $\text{C}{\text{s}}_{n\bar{v_{n}}}$ are the respective reduction (in percentage) in task communication between VMs located in the same and different nodes compared with task communication inside the same VM. To sum up, $\text{C}{\text{s}}_{nv_{n}}=1\geq \text{C}{\text{s}}_{n\bar{v_{n}}}\geq \text{C}{\text{s}}_{\bar{nv_{n}}}\geq 0$.

According to task communication, the idea is to add a component in the OF that penalizes (enhances) the communications performed between different VMs and different nodes. Grouping tasks inside the same VM will depend on not only their respective* processing cost* (*P*_*nv*_*n*__^*i*^) but also the* communication costs C*_*nv*_*n*__^*ij*^ and* communication slowdowns *Cs_*nv*_*n*__, $\text{C}{\text{s}}_{n\bar{v_{n}}}$, and $\text{C}{\text{s}}_{\bar{nv_{n}}}$. We reward the communications done in the same VM but less so the ones done in different VMs while still in the same node. Finally, communications between nodes are left untouched, without rewarding or penalizing.

In the same way, if we modelled the OF in function of the tak heterogeneity in (2), the communication component will be as follows (only the communication component of the OF is shown):

(8) max⁡⁡∑n=1N ∑vn=1Vn ‍∑i=1Ttnvni∑j<iTCnvnijtnvnjCsnvn+tnvn¯jCsnvn¯+tnvn¯jCsnvn¯.

And the OF function will be

(9) max⁡⁡∑n=1N∑vn=1Vn∑i=1TtnvniPnvni+∑j<iTCnvnijtnvnjCsnvn+tnvn¯jCsnvn¯+tnvn¯jCsnvn¯×ΔnvnE∑j=1TtnvnjPnvnj;α,λ.

### 3.6. Choosing SLA or Energy Saving

It is important to highlight that the optimization goal is two criteria (SLA and energy in our case). Thus, the user could prioritize the criteria. Assignments are performed starting from the most powerful VM. When this becomes saturated, task assignment continues with the next most powerful VM, regardless of the node it resides in. When this VM resides in another node (as in our case), the energy-saving criteria will be harmed. It would be interesting to provide a means of increasing criteria preferences in the model presented.

In order to highlight specific criteria (i.e., energy saving), one more additional component must be added to the OF. This component must enhance the assignment of tasks to the same node by assigning tasks to the most powerful nodes and not only to the most powerful VMs as before. This is the natural procedure to follow, because the OF is a maximum. Thus, the likelihood of less powerful nodes becoming idle increases and this gives the opportunity to power them off, hence saving energy.

The additional component can be defined in a similar way as for the relative* VM computing power* (Δ_*nv*_*n*__) of a VM_*nv*_*n*__. Instead, we obtain the relative* node computing power* of a Node_*n*_ (Θ_*n*_) as the normalized summatory of their forming VMs. Θ_*n*_ will inform about the computing power of Node_*n*_.

For *N* nodes, Θ_*n*_ is formally defined as

(10) $$\begin{array}{c} \Theta_{n}=\frac{\sum_{v_{n}=1}^{V_{n}}\Delta_{nv_{n}}}{\sum_{n=1}^{N}\sum_{v_{n}=1}^{V_{n}}\Delta_{nv_{n}}}, \end{array}$$

where ∑_*n*=1_^*N*^Θ_*n*_ = 1. To obtain Θ_*n*_, the parallel Linpack version (HPL: high performance Linpack) can be used. It is the one used to benchmark and rank supercomputers for the TOP500 list.

Depending on the importance of the energy saving criteria, a weighting factor should be provided to Θ_*n*_. We simply call this factor energy Energy Ξ. The Ξ will be in the range (0,…, 1]. For an Ξ0, our main criteria will be energy saving, and for Ξ = 1, our goal is only SLA. Thus, the resulting energy component will be Θ_*n*_Ξ. Thus, for a given Node_*n*_ with Θ_*n*_, we must weigh the energy saving criteria of such a node by the following factor:

(11) $$\begin{array}{c} \overset{V_{n}}{\underset{v_{n}=1}{\sum }}\;\overset{T}{\underset{i=1}{\sum }}t_{nv_{n}}^{i}\Theta_{n}\Xi . \end{array}$$

The resulting OF function will be

(12) max⁡⁡∑n=1N∑vn=1Vn ∑i=1TtnvniΘnΞ×∑vn=1Vn∑i=1TtnvniPnvni+∑j<iTCnvnijtnvnjCsnvn+tnvn¯jCsnvn¯+tnvn¯jCsnvn¯×ΔnvnE∑j=1TtnvnjPnvnj;α,λ.

### 3.7. Enforcing SLA

For either prioritized criteria, SLA or energy saving, there is a last consideration to be taken into account.

Imagine the case where tasks do not communicate. Once they are assigned to a node, one would expect them to be executed in the minimum time. In this case, there is already no need to group tasks in the VM in decreasing order of power in the same node, because this node is no longer eligible to be switched off. A better solution in this case would be to balance the tasks between the VMs of such a node in order to increase SLA performance. Note that this not apply in the communicating tasks due to the communication slowdown between VMs.

To implement this, we only need to assign every noncommunicating tasks without taking the* relative computing power* (Δ_*nv*_*n*__) of each VM into account.

We only need to replace Δ_*nv*_*n*__ in (12) by Δ, defined as

(13) Δ=if  ∑j<iTCnvnij≥0Δnvn else  1.

For the case of noncommunicating tasks, by assigning a Δ = 1, all the VMs have the same* relative computing power *Δ_*nv*_*n*__. Thus, tasks are assigned in a balanced way.

### 3.8. Model Formulation

Finally, the OF function and their constraints are presented. The best task scheduling assignment to VMs which takes all the features into account (*GS* policy) is formally defined by the following nonlinear programming model:

(14a) max⁡⁡∑n=1N∑vn=1Vn ∑i=1TtnvniΘnΞ×∑vn=1Vn∑i=1TtnvniPnvni+∑j<iTCnvnijtnvnjCsnvn+tnvn¯jCsnvn¯+tnvn¯jCsnvn¯×ΔE∑j=1TtnvnjPnvnj;α,λ

(14b)  s.t. ∑i=1TMnvni≤Mnvn ∀n≤N,  vn≤Vn

(14c)  ∑n=1N ∑vn=1Vtnvni=1 ∀i≤T.

Equation (14a) is the objective function (OF) to be maximized. Note that OF is an integer and nonlinear problem. Inequality in (14b) and equality in (14c) are the constraints of the objective function variables. Given the constants *T* (the total number of requests or tasks), and *M*_*v*_ for each VM_*nv*_*n*__, the solution that maximizes* OF* will obtain the values of the variables *t*_*nv*_*n*__^*i*^, representing the number of tasks assigned to VM_*nv*_*n*__. Thus, the *t*_*nv*_*n*_*i*_ obtained will be the assignment found by this model.

OF takes into account the* processing costs* (*P*_*nv*_*n*__^*i*^) and the* communication times* (*C*_*nv*_*n*__^*ij*^) of the tasks assigned to each VM_*nv*_*n*__ and the* communication slowdowns* between VMs $\text{C}{\text{s}}_{n\bar{v_{n}}}$ and nodes $\text{C}{\text{s}}_{\bar{nv_{n}}}$. Cs_*nv*_*n*__ = 1. Δ is defined in Section 3.7. And *E*(∑_*j*=1_^*T*^ *t*_*nv*_*n*__^*j*^ *P*_*nv*_*n*__^*j*^; *α*, *λ*) represents the power slowdown of each VM due to its workload (defined in Section 3.4).

To sum up, for the case when the workload is made up of noncommunicating tasks, if we are interested in prioritizing the SLA criteria, OF 3.5 should be applied. If, on the contrary, the goal is to prioritize energy saving, OF (14a) should be used instead.

## 4. Results

In this section, we present the theoretical results obtained from solving the scheduling problems aimed at achieving best task assignment. Two representative experiments were performed in order to test the performance of GS.

The experiments were performed by using the AMPL (AMPL. A Mathematical Programming Language. http://ampl.com) language and the SCIP (SCIP. Solving Constraint Integer Programs. http://scip.zib.de) solver. AMPL is an algebraic modeling language for describing and solving high-complexity problems for large-scale mathematical computation supported by many solvers. Integer and nonlinear (our model type) problems can be solved by SCIP, one of the solvers supported by AMPL.

Throughout all the experimentation, the* Erlang arguments* were obtained empirically by using the strategy explained in Section 3.4.

**Figure 2:**
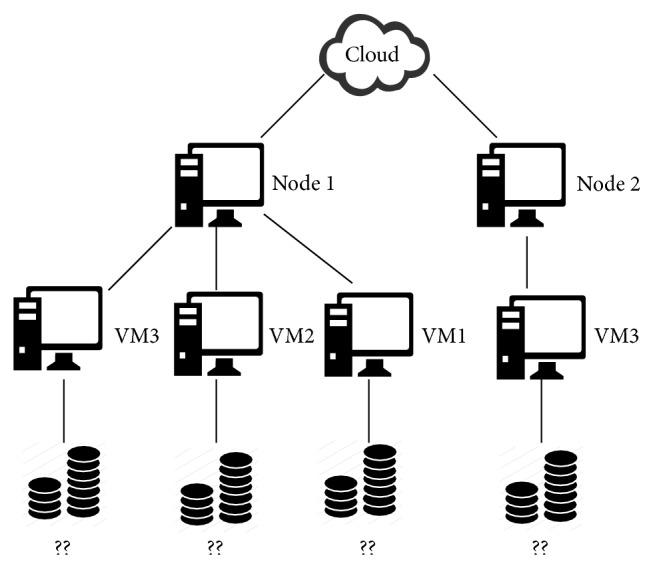
Cloud architecture.

As the objective of this section is to prove the correctness of the policy, only a small set of tasks, VMs, and nodes was chosen. The size of the experimental framework was chosen to be as much representative of actual cases as possible, but at the same time, simple enough to be used as an illustrative example. So, the experimental framework chosen was made up of 2 different nodes: one of them comprised 3 VMs and the other 1 VM; see Figure 2. The objective of this simulation was to achieve the best assignment for 3 tasks.  Table 1 shows the* processing cost P*_*nv*_*n*__^*i*^, relating the execution times of the tasks in each VM. To show the good behavior of the model presented, each task has the same* processing cost* independently of the VM. The model presented can be efficiently applied to real cloud environments. The only weak point is that the model is static. That means that homogenous and static workload conditions must be stable in our model. Job executions in different workload sizes can be saved in a database system, providing a means for determining the SLA of such a job in future executions.

### 4.1. Without Communications

In this section, a hypothetical situation without communication between tasks is evaluated. In this situation, our scheduling policy tends to assign the tasks to the most powerful set of virtual machines (i.e., with the higher* relative computing power *Δ_*nv*_*n*__, considering their individual saturation in this choice). This saturation becomes critical when more and more tasks are added. Here, the most important term is the* Erlang* function, since it models the behaviour of every virtual machine. Thus, taking this into account, our scheduler knows the exact weight of tasks it can assign to the VMs in order to obtain the best return times. This phenomenon is observed in the following examples.

#### 4.1.1. Without Communications and High Optimal* Erlang*

Table 2 shows the parameters used in the first example. The amount of* Memory* allocated to each task *t* ^*i*^ in every VM *M*_*nv*_*n*__ (as we supposed this amount to be equal in all the VMs, we simply call it *M* ^*i*^). The* relative computing power* (Δ_*nv*_*v*__) of each VM and finally the *α* and *λ Erlang arguments*. Note that all the VMs have the same* Erlang* parameters. The parameters were chosen this way because any VM saturates with the overall workload assigned. In other words, the total* processing cost* 7 is lower than the optimal* Erlang* workload 16.

Table 3 shows the solver assignment results. The best scheduling assigns all the tasks to the same VM (VM_21_, the only VM in node 2), because this VM has the biggest* relative computing power* (Δ_*nv*_*v*__). This result is very coherent. Due to the lack of communications, the model tends to assign tasks to the most powerful VM while its workload does not exceed the* Erlang* optimum (a workload of 10 tasks). As in our case, the total workload is 7, and VM_21_ could host even more tasks.

#### 4.1.2. Without Communications, Low Optimal* Erlang*, and Preserving SLA

In this example (see Table 4), the VMs have another* Erlang*. However, the* task processing costs* do not change, so they remain the same as in Table 1. In this case, each VM becomes saturated when the assignment workload weight is higher than 5 (because 5 is the optimal workload).

The best assignment in this case is the one formed by the minimum set of VMs with the best* relative computing power *Δ_*nv*_*v*__ (see Table 5 column Task Assignment SLA). The assignment of the overall tasks to only one VM (although it was the most powerful one) as before will decrease the return time excessively, due to its saturation.

#### 4.1.3. Without Communications, Low Optimal* Erlang*, and Preserving Energy Saving

Provided that the most important criterion is the energy saving, the assignment will be somewhat different (see Table 5, column Task Assignment Energy). In this case, OF (14a) with Ξ = 1 was used. Then, as expected, all the tasks were again assigned to VM_21_ of Node_2_.

### 4.2. With Communications

Starting from the same VM configuration shown on Table 4, in this section we present a more real situation where the costs of communications between tasks are also taken into account. It is important to highlight that in some situations, the best choice does not include the most powerful VMs (i.e., with the highest* relative computing power *Δ_*nv*_*n*__). Thus, the results shown in this section must show the tradeoff between* relative computing power* of VM, workload scheduling impact modeled by the* Erlang* distribution, and communication efficiency between tasks.

#### 4.2.1. High Communication Slowdown

This example shows the behaviour of the model under large communication costs between VMs (see Table 6). This table shows the* communication costs* between tasks (*C* ^*ij*^) and the* communication slowdown* when communications are done between VMs in the same node ($\text{C}{\text{s}}_{n\bar{v_{n}}}$) and the penalty cost when communications are performed between different nodes ($\text{C}{\text{s}}_{\bar{nv_{n}}}$). Note that the penalties are very high (0.2 and 0.1) when the communications are very influential.

The solver assignment is shown in Table 7. In order to avoid communication costs due to slowdowns, the best assignment tends to group tasks first in the same VM and second in the same node. Although VM_21_ should become saturated with this assignment, the high communication cost compensates the loss of SLA performance, allowing us to switch off node 1.

#### 4.2.2. Low Communication Slowdown

Now, this example shows the behaviour of our policy under more normal communication conditions. Here, the communication penalties between the different VMs or nodes are not as significant as in the previous case because $\text{C}{\text{s}}_{n\bar{v_{n}}}$ and $\text{C}{\text{s}}_{\bar{nv_{n}}}$ are higher.  Table 8 shows the communication costs between tasks and the penalty cost if communications are performed between VMs in the same node ($\text{C}{\text{s}}_{n\bar{v_{n}}}$) or between different nodes ($\text{C}{\text{s}}_{\bar{nv_{n}}}$).

In this case, the solver got as a result two hosting VMs (see Table 9) formed by the VM_11_ (with the assigned tasks *t* ^2^ and *t* ^3^) and VM_21_ with task *t* ^1^. In this case, due to the low differences between the different communication slowdowns, task assignment was distributed between the two nodes.

#### 4.2.3. Moderate Communication Slowdown

We simulated a more normal situation, where the communication slowdown between nodes is higher than the other ones. From the same example, it was only reduced $\text{C}{\text{s}}_{\bar{nv_{n}}}$ to 0.4 (see Table 10). As expected, the resulting solver assignment was different from that in the previous case. Theoretically, this assignment should assign tasks to the powerful unsaturated VMs, but as much as possible to the VMs residing on the same node. The solver result was exactly what was expected. Although the most powerful VM is in node 2, the tasks were assigned to the VMs of node 1, because they all fit in the same node. That is the reason why a less powerful node like node 1, but one with more capacity, is able to allocate more tasks than node 2 due to the communication slowdown between nodes. These results are shown in Table 11.

## 5. Conclusions and Future Work

This paper presents a cloud-based system scheduling mechanism called GS that is able to comply with low power consumption and SLA agreements. The complexity of the model developed was increased, thus adding more factors to be taken into account. The model was also tested using the AMPL modelling language and the SCIP optimizer. The results obtained proved consistent over a range of scenarios. In all the cases, the experiments showed that all the tasks were assigned to the most powerful subset of virtual machines by keeping the subset size to the minimum.

Although our proposals still have to be tested in real scenarios, these preliminary results corroborate their usefulness.

Our efforts are directed towards implementing those strategies in a real cloud environment, like the OpenStack [22] or OpenNebula [23] frameworks.

In the longer term, we consider using some statistical method to find an accurate approximation of the workload using well-suited* Erlang* distribution functions.

## Figures and Tables

**Table 1:** Task processing costs.

Task *t* ^*i*^	Processing costs *P*_*nv*_*n*__^*i*^	Value
*t* ^1^	*P*_11_^1^, *P*_12_^1^, *P*_13_^1^, *P*_21_^1^	**1**
*t* ^2^	*P*_1_^2^, *P*_12_^2^, *P*_13_^2^, *P*_21_^2^	**5**
*t* ^3^	*P*_11_^3^, *P*_12_^3^, *P*_13_^3^, *P*_21_^3^	**1**

Total processing cost (∑_*i*_‍*P*_*nv*_*n*__^*i*^)		**7**

**Table 2:** Without communications. High optimal *Erlang*. VM configurations.

Node	VM_*nv*_*n*__	*M* ^*i*^	Δ_*nv*_*v*__	*Erlang *
**1**	VM_11_	**10**	**0.75**	*α* = 3, *λ* = 8
**1**	VM_12_	**10**	**0.35**	*α* = 3, *λ* = 8
**1**	VM_13_	**10**	**0.1**	*α* = 3, *λ* = 8
**2**	VM_21_	**10**	**0.85**	*α* = 3, *λ* = 8

**Table 3:** Without communications. High optimal *Erlang*. Solver assignment.

Node	VM_*nv*_*n*__	Task assignment
**1**	VM_11_	**0**
**1**	VM_12_	**0**
**1**	VM_13_	**0**
**2**	VM_21_	*t* ^1^, *t* ^2^, *t* ^3^

**Table 4:** Without communications. Low optimal *Erlang*. Preserving SLA. VM configurations.

Node	VM_*nv*_*n*__	*M* ^*i*^	Δ_*nv*_*v*__	*Erlang *
**1**	VM_11_	**10**	**0.75**	*α* = 5, *λ* = 1
**1**	VM_12_	**10**	**0.35**	*α* = 5, *λ* = 1
**1**	VM_13_	**10**	**0.1**	*α* = 5, *λ* = 1
**2**	VM_21_	**10**	**0.85**	*α* = 5, *λ* = 1

**Table 5:** Without communications. Low optimal *Erlang*. Preserving SLA. Solver assignment.

Node	VM_*nv*_*n*__	SLA	Energy
		Task assignment	Task assignment
**1**	VM_11_	*t* ^1^, *t* ^3^	*t* ^2^
**1**	VM_12_	**0**	*t* ^1^, *t* ^3^
**1**	VM_13_	**0**	**0**
**2**	VM_21_	*t* ^2^	**0**

**Table 6:** High slowdown. Communication configurations.

Task *t* ^*i*^	Task *t* ^*j*^	Communication costs *C* ^*ij*^
*t* ^1^	*t* ^2^	**0.2**
*t* ^1^	*t* ^3^	**0.6**
*t* ^2^	*t* ^3^	**0.3**
$Cs_{n\bar{v_{n}}}$		**0.2**
$Cs_{\bar{nv_{n}}}$		**0.1**

**Table 7:** High slowdown. Solver assignment.

Node	VM_*nv*_*n*__	Task assignment
**1**	VM_11_	**0**
**1**	VM_12_	**0**
**1**	VM_13_	**0**
**2**	VM_21_	*t* ^1^, *t* ^2^, *t* ^3^

**Table 8:** Low slowdown. Communication configurations.

Task t^i^	Task t^j^	Communication costs C^ij^
*t* ^1^	*t* ^2^	**0.2**
*t* ^1^	*t* ^3^	**0.6**
*t* ^2^	*t* ^3^	**0.3**
$Cs_{n\bar{v_{n}}}$		**0.9**
$Cs_{\bar{nv_{n}}}$		**0.8**

**Table 9:** Low slowdown. Solver assignment.

Node	VM_*nv*_*n*__	Task assignment
**1**	VM_11_	*t* ^1^, *t* ^3^
**1**	VM_12_	**0**
**1**	VM_13_	**0**
**2**	VM_21_	*t* ^2^

**Table 10:** Moderate slowdown. Communication configurations.

Task *t* ^*i*^	Task *t* ^*j*^	Communication costs *C* ^*ij*^
*t* ^1^	*t* ^2^	**0.2**
*t* ^1^	*t* ^3^	**0.6**
*t* ^2^	*t* ^3^	**0.3**
$Cs_{n\bar{v_{n}}}$		**0.9**
$Cs_{\bar{nv_{n}}}$		**0.4**

**Table 11:** Moderate slowdown. Solver assignment.

Node	VM_*nv*_*n*__	Tasks assignment
**1**	VM_11_	*t* ^2^, *t* ^3^
**1**	VM_12_	*t* ^1^
**1**	VM_13_	**0**
**2**	VM_21_	**0**
